# Radiation therapy for older patients with brain tumors

**DOI:** 10.1186/s13014-017-0841-9

**Published:** 2017-06-19

**Authors:** Giuseppe Minniti, Andrea Riccardo Filippi, Mattia Falchetto Osti, Umberto Ricardi

**Affiliations:** 1 Department of Neurological Sciences, IRCCS Neuromed, Via Atinense, Pozzilli, (IS) Italy; 2 UPMC San Pietro FBF, Radiotherapy Center, Rome, Italy; 3 Department of Oncology, University of Torino, Torino, Italy; 4 Radiation Oncology Unit, S Andrea Hospital, University Sapienza, Rome, Italy

**Keywords:** Brain tumors, Elderly, Radiotherapy, Chemotherapy, Radiosurgery

## Abstract

The incidence of brain tumors in the elderly population has increased over the last few decades. Current treatment includes surgery, radiotherapy and chemotherapy, but the optimal management of older patients with brain tumors remains a matter of debate, since aggressive radiation treatments in this population may be associated with high risks of neurological toxicity and deterioration of quality of life. For such patients, a careful clinical status assessment is mandatory both for clinical decision making and for designing randomized trials to adequately evaluate the optimal combination of radiotherapy and chemotherapy.

Several randomized studies have demonstrated the efficacy and safety of chemotherapy for patients with glioblastoma or lymphoma; however, the use of radiotherapy given in association with chemotherapy or as salvage therapy remains an effective treatment option associated with survival benefit. Stereotactic techniques are increasingly used for the treatment of patients with brain metastases and benign tumors, including pituitary adenomas, meningiomas and acoustic neuromas. Although no randomized trials have proven the superiority of SRS over other radiation techniques in older patients with brain metastases or benign brain tumors, data extracted from recent randomized studies and large retrospective series suggest that SRS is an effective approach in such patients associated with survival advantages and toxicity profile similar to those observed in young adults. Future trials need to investigate the optimal radiation techniques and dose/fractionation schedules in older patients with brain tumors with regard to clinical outcomes, neurocognitive function, and quality of life.

## Introduction

Cancer is most frequently diagnosed among individuals aged 65 years and older [1–3], and the number of older patients with cancer will increase in the future as result of increasing life expectancy of the population [4]. As for other cancers, the incidence of either malignant or benign brain tumors has been increasing in the elderly population [4], representing an important aspect of public health.

Radiotherapy (RT) given alone or in combination with systemic therapy is a cornerstone of the multidisciplinary management of brain tumors and remains an attractive option for older patients [5]. Advances in radiation planning and dose delivery have improved the safety and efficacy of RT, although irradiation of brain tumors is particularly challenging in older patients because of the potential increased radiation-induced toxicity secondary to comorbidities, impaired functional status and neurocognitive function. In addition, older patients are under-represented in randomized controlled clinical trials and clinicians need to extrapolate data from studies done with a much younger cohort. However, treating older patients is not the same as treating patients in their 50s or 60s. The clinical behavior of some tumors changes with age. Some become more aggressive due to a high prevalence of unfavorable genomic changes or resistance to chemotherapy. For these reasons, treatment paradigms for older patients with brain tumors are not well defined.

The purpose of this review is to summarize the published literature on the clinical outcomes of RT for the most common brain tumors in the elderly population, and to address important issues such as optimal radiation dose and fractionation, combining RT with systemic therapy, quality of life and neurocognitive function after RT, and future research priorities for this population.

## Methods

A literature search was conducted in MEDLINE PubMed evaluating older people with brain tumors. The search focused on randomized, prospective and retrospective studies published in English. The literature search was performed using a combination of medical subject headings (MeSH) (“brain tumors/radiotherapy” or “gliomas” or “brain metastases” or “lymphomas” or “meningiomas” or “pituitary adenomas” or “acoustic neuromas” or “older” or “elderly”) and free text terms (“toxicity” or “radiosurgery” or “fractionated stereotactic radiotherapy” or “chemotherapy” or “chemoradiation”). Relevant prospective and retrospective studies published from 1990 to 2017 were included. Studies published in languages other than English or not involving human subjects were not reviewed. There was no definitive age cutoff used for defining older patients. A total of 312 potentially relevant studies were identified, including 47 prospective/randomized studies and 265 retrospective studies. The results of the literature research were used and included if appropriate.

## General aspects of radiation treatment in older patients

The aging process is characterized by a decrease in the function of various organ systems, as well functional, cognitive, emotional, and socioeconomic changes [6, 7]. It is also associated with an increased incidence of comorbidities and geriatric syndromes. Common geriatric syndromes include delirium, gait imbalance, malnutrition, and incontinence that can complicate treatments and may increase patient morbidity [8]. When considering the appropriate therapy for an older patient with cancer, a baseline assessment of these multiple factors can be useful to determine if a patient is fit or frail [9–11]. Fit older adults have few comorbidities, no functional deficits, any or few geriatric syndromes, and generally may be considered appropriate for the same therapies used in younger adults. In contrast, frail patients have difficulty of maintaining functional independence, multiple chronic conditions and geriatric syndromes that make them more vulnerable to toxicities from therapy. In order to help cancer specialists to determine the best treatment for their older patients, the U.S. National Comprehensive Cancer Network, the European Society of Breast Cancer Specialists, the International Society of Geriatric Oncology (SIOG), and the European Organization for the Research and Treatment of Cancer have recommended the use of a comprehensive geriatric assessment (GA) in older patients with cancer [12–16].

A GA is a multidisciplinary diagnostic process that evaluates the risk of adverse outcomes of death and functional decline in older cancer patients. A comprehensive GA includes the evaluation of functional status, cognitive function, nutritional status, comorbidities, polipharmacy, and socioeconomic status in every older cancer patient with the aim of developing the optimal treatment plan. Several systematic reviews have showed that GA is beneficial in improving outcome and reducing the risk of adverse outcomes [17–19].

Several instruments have been reported in the literature to assess the different domains of GA [6, 20, 21–37] (Table 1). Functional assessment instruments, such as Eastern Cooperative Oncology Group (ECOG) performance status scale and the Karnofsky Performance Scale (KPS) are widely used in oncology setting. Additional instruments to assess the functional status that capture additional information not obtained by accessing performance status alone include activities of daily living (ADLs) and instrumental activities of daily living (IADLs) [6, 22, 23]. ADLs are a measure of six basic self-care skills: bathing, dressing, toileting, continence, tranferring, and feeding [6]; IADLs refers to 8 functions that are needed to live independent and include travelling, shopping, using the phone, preparing meals, laundry, doing house-work, taking medicine and managing money [22]. Cognitive function may be assessed by several instruments, including the Mini-Mental State Examination (MMSE) [24], the Montreal Cognitive Assessment (MoCA) [25], the Blessed Orientation Memory Concentration [26], and the Clock drawing test [27]. Comorbidities that can have significant impact on treatment tolerance and outcome are usually measured by standardized instruments as the Charlson Comorbidity Index (CGI) [29] and the Cumulative Illness Rating Scale-Geriatrics (CIRS-G) [30]. Additional validated tools included in GA explore depression and psychological distress (Geriatric depression scale [31], hospitalized anxiety and depression scale [32]; patient health questionnaire [33, 34], nutrition status (body mass index; Mini Nutritional Assessment [35], and socioeconomic status [38, 39]. Currently, no specific tools are usually recommended and their choice depends both on the resource available at different cancer centers and patient population.

**Table 1:** Domains of geriatric assessment and examples of instruments used for each domain

Domain	Commonly used instruments
Physical function/falls [20, 21]	Timed up and go [20]
	Self-reported number of falls
	Short Physical performance battery [21]
	Grip strenght
Functional status [6, 22]	Activities of daily living (ADLs)
	Instrumental activities of daily living (IADLs)
Cognitive function [23–28]	Mini Mental State Examination
	Montreal cognitive Assessment
	Blessed Orientation Memory Concentration Test
	Clock Drawing Test
	Memorial Delirium Assessment Scale
Comorbidities [29, 30]	Charlson Comorbidity Index (CGI)
	Cumulative Illness Rating Scale-Geriatrics (CIRS-G)
Depression/Psycological status [31, 34]	Geriatric depression scale
	hospitalized anxiety and depression scale
	patient health questionnaire
Nutrion [35]	Body mass index
	Mini Nutritional Assessment Short Form
Polipharmacy [36, 37]	Medication Appropriateness index
	STOPP/START Criteria
Socioeconomic status [38, 39]	Lubben Social Network Scale

A full comprehensive GA takes an average between 30 and 120 min and this may limit its use in all older cancer patients in a busy clinical practice. Thus, several screening tests have been developed and implemented in daily practice. The most widely used screening instruments are the G8 [40], the abbreviated comprehensive geriatric assessment (aCGA) [41], the Groningen frailty indicator (GFI) [42], and the vulnerable elders survey-13 (VES-13) [43]. In general, those older adults who scored above the cutoff of the screening instruments should receive a complete GA.

In summary, GA is a critical process that can help to identify fit, vulnerable, or frail older cancer patients. Its use should be implemented in clinical practice to help oncologists to guide cancer treatment decision-making and improve function and quality of life of older patients. GA models need to be incorporated in future clinical trials in order to validate their effectiveness in older patients with brain cancer treated with RT and/or chemotherapy.

## Malignant gliomas

Glioblastoma (GBM) is the most common primary brain tumor in adults, with an incidence rate among elderly patients of 70 years and older of 17.5 per 100,000 person-years, and a relative risk of 3–4 times compared with young adults [4].

RT is frequently used in older patients with GBM. Its superiority over supportive care alone has been demonstrated in a French multi-institutional randomized trial of 85 elderly patients aged 70 years and older [44]. The median survival and progression-free survival times were 29.1 and 14.9 weeks in patients treated with RT (50 Gy given in daily fractions of 1.8 Gy) plus supportive care, and 16.9 and 5.4 weeks for those treated with supportive care alone (*p* = 0.002), respectively. As compared with supportive care, RT did not cause further deterioration in KPS, quality of life and cognitive function.

The efficacy of either radical RT or abbreviated courses of RT has been evaluated in randomized and prospective studies [45–54] (Table 2). Roa et al. [50] have conducted a trial of patients aged 60 years and older with newly diagnosed GBM randomized to receive standard RT (60 Gy in 30 daily fractions) or an abbreviated course of RT (40 Gy in 15 daily fractions) after surgery. The median survival time and 6-month survival rates were similar between groups, being 5.1 months and 44.7% after standard RT and 5.6 months and 41.7% after short-term RT (*p* = 0.57), respectively. In another study of 291 patients older than 60 years with newly diagnosed GBM (Nordic study) randomly assigned to receive RT or chemotherapy with temozolomide (TMZ), RT was given as radical RT or hypofractionated RT (34 Gy given in 3.4 Gy fractions over 2 weeks). Median overall survival was significantly longer for patients who received TMZ than those who received standard RT (8.3 months vs 6.0 months, hazard ratio 0.7, 95% CI 0.52–0.93, *p* = 0.01), but not significantly better than those treated with hypofractionated RT (7.5 months, HR 0.82, 95% CI 0.63–1.06, *p* = 0.12); for patients older than 70 years, median overall survival was better with hypofractionated RT than with standard RT (7.0 months vs 5.2 months, *p* = 0.02).

**Table 2:** Selected published studies on radiotherapy or chemotherapy in older patients with high.grade gliomas/glioblastomas

Authors	Type of study	Patients	Age years	RT dose Gy/fractions	CHT	Median PFS months	Median OS months
Bauman GS et al. [45]	Prospective	29	≥65	30/10	no	NA	6
Ford JM et al. [46]	Prospective	27	≥60	36/12	no	NA	4 (11% at 1 year)
Hoegler DB et al. [47]	Prospective	25^a^	≥70	37.5/15	no	NA	8
McAleese JJ et al. [48]	Prospective	30 29	65-70 ≥ 70	30/6 30/6	no no	NA NA	37% at 6 months 41% at 6 months
Chinot O et al. [49]	Prospective	32	≥70	no	TMZ°	5 (15% at 1 year)	6.4 (25% at 1 years)
Roa W et al. [50]	Randomized	51 49	≥60 ≥ 60	60/30 40/15	no no	NA NA	5.1 5.6
Keime-Guiber F et al. [44]	Randomized	39 39	≥70 ≥ 70	50/28 no	no no	3.6 1.5	7 4
Gallego Perez-Larraya et al. [51]	Prospective	70	≥70	no	TMZ°	4 (6.5% at at 1 year)	6 (11.4% at 1 year)
Malmstrom et al. [52]	Randomized	100 98 93	>60 > 60	60/30 34/10 no	no no TMZ°	NA NA NA	6 (17% at 1 year) 7.5 (23% at 1 year) 8.3 (27% at 1 year)
Wick et al. [53]	Randomized	178^a^ 195^a^	>65 > 65	60/30 no	no TMZ °°	4.7 (9.3% at 1 year) 3.3 (12% at 1 year)	9.6 (37.4% at 1 year) 8.6 (34.4% at 1 year)
Roa et al. [54]	Randomized	48^a^ 50^a^	≥65 ≥ 65	40/15 25/5	no no	4.2 4.2	7.9 6.4

*RT* radiotherapy, *CHT* chemotherapy, *OS* overall survival, *PFS* progression-free survival, *PCV* procarbazine, *CCNU* Vincristine, *TMZ* Temozolomide, TMZ (200 mg/m2 on days 1–5) every 4 weeks, *TMZ* week-on/week-off (100 mg/m2 on days 1–7)

^a^Series include both elderly and frail patients, *NA*, not assessed

In a recent Canadian study of 98 frail and/or elderly patients with GBM randomized to receive two different hypofractionated radiation schedules, Roa et al. [54] observed median overall survival times of 7.9 months (95% CI, 6.3 to 9.6 months) in patients who received 25 Gy in five daily fractions and 6.4 months (95% CI, 5.1 to 7.6 months) in those receiving 40 Gy in 15 daily fractions over 3 weeks (*p* = 0.9), with a similar median progression-free survival times of 4.2 months in both groups. With a median follow-up time of 6.3 months, the quality of life between groups at 4 weeks and 8 weeks after treatment was not different.

The main concern about the use of a radical course of RT in older patients with GBM is the potential high incidence of radiation-induced neurological toxicity and deterioration of quality of life. Roa et al. [50] reported no significant differences in KPS scores over time between standard RT and hypofractionated RT, although 49% of patients treated with standard RT required an increase in corticosteroid dosage as compared with 23% of patients who received short-term RT (*p* = 0.02). Similarly, in the Nordic study no significant differences were observed in physical, role, emotional, social, and cognitive functioning, and global health status between patients receiving standard RT or hypofractionated RT [52]. However, data should be interpreted with caution because of the low number of completed questionnaires. In a prospective series of 43 elderly patients aged 70 years and older with GBM who received hypofractionated RT given at the dose of 30 Gy in 6 fractions over 2 weeks followed by adjuvant TMZ, no negative effects of treatment on KPS and health-related quality of life scores were observed [55]. Analysis of the European Organisation for Research and Treatment of Cancer (EORTC) quality of life (QOL) C-30 questionnaires showed that global health status and several functioning scales, including physical, role, emotional, social, and cognitive functioning, did not deteriorate in the majority of patients until tumor progression. An improved functional status has been reported by others using other hypofractionated schedules [45, 48].

The use of chemotherapy as an alternative to RT in older patients with malignant gliomas has been addressed in a few prospective and randomized studies [49, 51–53] (Table 2). In the German Neuro-oncology Working Group (NOA) phase 3 trial (NOA-08), 373 patients older than 65 years with histologically confirmed anaplastic astrocytoma or GBM, and a KPS score ≥ 60, were randomly assigned to receive dose-dense TMZ (1 week on, 1 week off schedule, 100 mg/m^2^ given on days 1–7) or standard RT [53]. Median event-free survival time was 3.3 months for patients treated with TMZ and 4.7 months for those treated with standard RT (hazard ratio 1.15, 95% CI 0.92–1.43, *p* = 0.03), respectively. Median survival was 8.6 months for patients treated with TMZ and 9.6 months for those treated with standard RT (hazard ratio 1.09, 95% CI 0.84–1.42, *p* = 0.03), respectively, indicating that chemotherapy was non-inferior to standard RT. Analysis of health-related quality of life scales showed no significant differences between groups; however, grade 2–4 adverse events were more frequent in the TMZ group. A striking finding of the study was the predictive role of O6-methylguanin-DNA-methyltransferase (MGMT) promoter methylation status on survival outcomes. MGMT promoter methylation was associated with longer survival (median 11.9 months vs 8.2 months; hazard ratio 0.62, 95% CI 0.42–0.91, *p* = 0.014) and longer event-free survival (median 5.7 months vs 3.5 months; hazard ratio 0.5, CI 0.36–0.68, *p* < 0.001) than unmethylated status. The presence of MGMT promoter methylation was associated with better event-free survival time only in patients who received TMZ, but not in patients who received RT, whereas the opposite was true for patients with unmethylated tumor.

In the Nordic study [52], no survival differences were observed amongst patients aged 60–70 years who received standard RT, hypofractionated RT or TMZ; however, for patients older than 70 years median overall survival was better with TMZ and hypofractionated RT than with standard RT (9.0 and 7.0 months vs 5.2 months, *p* < 0.0001 and *p* = 0.02). Data for health-related quality of life, including cognitive functioning and global health status, were generally better in patients who received TMZ than in those who received RT; however, because of the low number of completed questionnaires results need to be interpreted with caution. As for the NOA-8 trial, the study confirmed the predictive value of MGMT promoter methylation. Amongst patients treated with TMZ, median overall survival was 9.7 months for those with methylated tumors and 6.8 for those with unmethylated tumors (*p* = 0.02). In contrast, MGMT methylation status did not affect survival in patients treated with RT (8.2 months in methylated vs 7.0 months in unmethylated tumors, *p* = 0.81).

The use of standard or hypofractionated RT in combination with adjuvant and/or concomitant TMZ, which is the standard treatment in adult patients with GBM, has been reported in several studies [56–67]; selected prospective series are showed in Table 3. In a small prospective series of 32 patients aged 70 years and older treated with radical RT in combination with adjuvant and concomitant TMZ at University of Rome Sapienza, Sant’Andrea Hospital, the reported median overall survival and 1-year survival rates were 10.6 months and 37%, respectively [58]. Grade 3 or 4 hematologic toxic effects occurred in 24% of patients. Brandes et al. [59] have reported the results of 58 patients aged 65 years and older who received standard chemoradiation for a newly diagnosed GBM. The overall median survival was 13.7 months, being significantly better in patients with methylated tumors (*p* = 0.05). Overall survival rates were 83 and 69% for patients with methylated tumors and 56 and 38% for those with unmethylated tumors at 2 and 3 years, respectively. Radiation Therapy Oncology Group (RTOG) Grade 2 and grade 3 mental status deterioration were detected in 31 and 25% of patients, respectively.

**Table 3:** Selected studies on combined radiochemotherapy in older patients with glioblastoma

Authors	Type of study	Patients	Age years	RT dose Gy/fractions	CHT	median PFS months	median OS months
Brandes et al. [56]	Prospective	24 32 22	≥65 ≥ 65 ≥ 65	59.4/33 59.4/33 59.4/33	no PCV TMZ	5.3 (8.3% at 1 year) 6.9 (15.6% at 1 year) 10.7 (47.4% at 1 year)	11.2 (31.6% at 1 year) 12.7 (56.2% at 1 year) 14.9 (72.5% at 1 year)
Minniti G et al. [58]	Prospective	32	≥70	60/30	TMZ	6.7 (16% at 1 year)	10.8 (37% at 1 year)
Brandes et al. [59]	Prospective	58	≥65	60/30	TMZ	9.5 (35% at 1 year)	13.7 (31.4% at 2 years)
Minniti G et al. [61]	Prospective	43	≥70	30/6	TMZ	6.3 (12% at 1 year)	9.3 (35% at 1 year)
Minniti et al. [66]	Prospective	70	≥70	40/15	TMZ	6 (20% at 1 years	12.4 (58% at 1 year)
Perry et al. [67]	Randomized	178^a^ 195^a^	>65 > 65	40/15 40/15	no TMZ	4.7 (9.3% at 1 year) 3.3 (12% at 1 year)	9.6 (37.4% at 1 year) 8.6 (34.4% at 1 year)

*RT* radiotherapy, *CHT* chemotherapy, *OS* overall survival, *PFS* progression-free survival, *PCV* procarbazine, *CCNU* Vincristine, *TMZ* temozolomide given concomitantly (75 mg/m2/day) and adjuvantly (200 mg/m2 on days 1–5 every four weeks)

^a^series include anaplastic astrocytomas and glioblastomas

The use of hypofractionated RT using a dose of 40 Gy given in 15 daily fractions in association with concomitant and adjuvant TMZ has been evaluated in a phase 2 trial in 70 patients aged 70 years and older with newly diagnosed GBM [63]. The median overall survival time and 1-year survival rate were 12.4 months and 58%; respective progression-free survivals were 6 months and 20%. MGMT promoter methylation was the most significant favorable prognostic factor for survival. The 1-year and 2-year survival rates were 81 and 20% in MGMT methylated tumors, and 32 and 0% in MGMT unmethylated tumors, respectively (*p* = 0.0001). The treatment was well tolerated and was consistently associated with improvement or stability in most of health-related quality of life scales [66]. Global health, social functioning, and cognitive functioning scores improved significantly between baseline and 6-month follow-up.

Results of the intergroup EORTC 26062-22061-NCIC Clinical Trials Group (NCI-CTG) CE6 randomized trial comparing the same regimen of hypofractionated RT (40 Gy in 15 fractions) to hypofractionated RT plus concomitant and adjuvant TMZ in 562 patients older than 65 years old with newly diagnosed GBM have been recently published [67]. RT plus TMZ significantly improved overall survival time (9.3 vs 7.6 months, *p* < 0.0001) and progression-free survival (5.3 vs 3.9 months, *p* < 0.0001) over RT alone. MGMT methylation status was the strongest prognostic factor for survival. Amongs 165 patients with methylated MGMT status, overall survival was 13.5 months and 7.7 months in RT + TMZ group and RT group, respectively (*p* = 0.0001); in unmethylated patients, respective overall survival was 10.0 months and 7.9 months (*p* = 0.055). Analyses of quality of life assessed by the EORTC Quality of Life Questionnaire–Core 30 (QLQ-C30) and the EORTC brain module (QLQ-BN20) showed that nausea and constipation were worse during chemoradiotherapy than during RT alone, but changes in the scores of all other symptom and function domains were similar in the two groups. In a large retrospective study of 243 older patients with GBM of 65 years or older who received standard RT or short-course RT plus concomitant and adjuvant temozolomide, the two treatments resulted in similar survival benefits of about 12 months, although short-course RT was associated with lower risks of neurological deterioration.

In summary, RT remains an essential treatment options in older patients with newly diagnosed GBM. An abbreviated course of RT may provide survival benefits similar to those reported with radical RT, maintaining an acceptable quality of life and potentially avoiding the long-term toxicity of more aggressive treatments. TMZ may represent a reasonable treatment option in older patients with MGMT promoter methylated tumors that is associated with survival benefit similar or even better than that reported with standard RT. In contrast, TMZ produces no benefit in patients with unmethylated tumors, and its use as initial treatment is not recommended. Recent studies have clearly demonstrated that the addition of concomitant and adjuvant TMZ to an abbreviated course of RT is a safe and more effective treatment for older patients with GBM as compared with RT alone, suggesting that chemoradiation can be considered the standard therapeutic option for this population.

## Primary central nervous system lymphoma

The incidence of primary central nervous system lymphoma (PCNSL), a lymphoproliferative disorder that may affect the brain, eyes, spinal cord or leptomeninges in absence of systemic involvement, is 3–4% of all primary brain tumors [68]. PCNSL has a predilection for the elderly population, with a median age at diagnosis of 55 years and a peak incidence in the sixth and seventh decades of life [69, 70]. Whole brain radiation therapy (WBRT) was historically the modality of choice to treat PCNSL until the early 1990s. PCNSL responds relatively quickly to RT, and the complete disappearance of enhancing tumor masses is frequently observed. However, local recurrence and intracranial progression at distant brain sites occurs within few months, and the reported survival outcome of patients treated by radiation alone is relatively poor [71, 72].

A combination of chemotherapy and WBRT has been evaluated in several studies in order to improve the disappointing survival observed after WBRT alone. High-dose methotrexate (hd-MTX) is currently the cornerstone of treatment of PCNSL with or without consolidation WBRT [73–75]; RT is typically given at 36–45 Gy in 1.6–1.8 Gy/fraction, with the aim to delay progression and improve survival. The superiority of combination of hd-MTX and radiation over radiation alone has been observed in several studies [76–78], even if a formal comparison has never been carried out; however, combined modality therapy appears to be associated with an increased risk of neurotoxicity, and its use has been questioned particularly in older patients [79, 80]. In a study of 31 patients with PCNSL treated with hd-MTX, WBRT, and high-dose cytarabine, Abrey et al. [79] observed an incidence of severe late treatment-related toxicity in nearly one third of patients, with those of 60 years and older of age at higher risk (*p* < 0.0001).

The use of chemotherapy alone for older patients with PCNSL, with the omission of consolidation RT, the standard approach until recent years, has therefore been tested in prospective studies over the past 2 decades [81–88]. In the German G-PCNSL-SG-1 randomized trial [81], 318 patients with a median age of 63 years who received primary treatment with hd-MTX-containing chemotherapy were then randomized to receive WBRT or not. The median overall survival was 32.4 months among patients that received WBRT, and 37.1 months in patients treated with chemotherapy alone (*p* = 0.71). Although the study failed to show the non-inferiority of omitting the WBRT, there were strong indications that this was the case for older patients [88] (Table 4). In addition, older patients or patients with a poor performance status who received WBRT experienced a higher incidence of severe neurotoxicity. Survival times of 14 to 36 months have been reported in other published studies with the use of MTX given in combination with other systemic agents including procarbazine, vincristine, cytarabine, temozolomide, rituximab, and lomustine [82–87] (Table 4).

**Table 4:** Selected prospective studies on radio/chemotherapy in older patients with primary central nervous system lymphoma (PCNSL)

Authors	Type of study	Patients	Age years	CHT	RT	Median PFS months	Median OS months
O’Neill et al. [81]	single arm phase II	21	>60	CHOP and HD-ARAC	50,4 Gy WB	6.2, 25% at 1 year	8, 14% at 2 year
Fritsch et al. [82]	single arm phase II	28	>65	R-MCP	No RT	31% at 3 years	31% at 3 years
Ghesquières et al. [83]	multicenter phase II	54	61-70 > 70	age-adapted C5R	20 Gy WB 30 Gy boost	61-70, 2% at 5 years >70, 11% at 5 years	61-70, 31% at 5 years >70, 17% at 5 years
Hoang-Xuan et al. [84]	multicenter phase II	50	>60	hd-MTX, lomustine, procarbazine, intrathecal MTX and ARA-C	No RT	40% at 1 year	14.3
Illerhaus G et al. [85]	multicenter phase II	31	>65	hd-MTX, procarbazine, CCNU	50 Gy WB for not responders	5.9	15.4 (33% at 5 years)
Laack et al. [86]	multicenter phase II	19	>70	HD-metilprednisolone	41.4 Gy WB 9 Gy boost	3.4, 32% at 6 months	5.5, 37% at 6 months
Roth P et al. [87]	multicenter phase II (G-PCNSL-SG-1)	66	>70	HD-MTX	+/− RT	4 (16.1 for complete responders)	12.5

*RT* radiotherapy, *CHT* chemotherapy, *OS* overall survival, *PFS* progression-free survival, *WB* whole brain, *CHOP* cyclophosphamide-adriamycin-vincristine-prednisone, *HD-ARAC* postirradiation high-dose cytarabine, *R-MCP* rituximab, methotrexate, procarbazine, lomustine, *C5R* methotrexate, doxorubicin, vincristine, cyclophosphamide, cytarabine, *hd-MTX* high dose methotrexate, *CCNU* lomustine

While these studies indicate that WBRT can be withheld and a radiological surveillance policy adopted for older patients who achieve complete remission, RT should be considered for those with residual disease after chemotherapy or in patients whose medical comorbidity precludes chemotherapy. An alternative approach to standard WBRT in older patients is represented by the use of low-dose consolidative RT, in the effort of maintaining the potential benefit of radiation while limiting the risk of neurotoxicity [89, 90]. In a phase II study conducted at Memorial Sloan Kettering, 52 patients were treated with WBRT, using 23.4 Gy in 1.8 Gy fractions after hd-MTX, rituximab, vincristine and procarbazine, and 2 cycles of consolidative high-dose Ara-C. Results, with rigorous neurocognitive testing, showed very good disease control (35% of patients relapsed) with minimal neurotoxicity [89]. In another retrospective series of 33 patients with PCNSL who received consolidation WBRT after HD-MTX, Ferreri et al. [90] observed no significant difference in disease control between patients who received WBRT doses ≥40 Gy or doses of 30–36 Gy (relapse rate, 46 vs. 30%; 5-year failure-free survival, 51 vs. 50%; *p* = 0.26). Currently, the randomized phase II study RTOG 1114 is exploring the effects of rituximab, methotrexate, procarbazine, vincristine and cytarabine with and without low-Dose WBRT for PCNSL. A few series suggest that the use of partial brain irradiation may be considered in patients with a single tumor [72, 91]; however, in current clinical practice WBRT remains the standard technical approach (including optic nerves and with a lower limit at C1-C2).

In summary, most studies support the use of chemotherapy-only treatments for elderly patients given the high risks of neurotoxicity associated with radiotherapy. Despite the concerns about the detrimental neurocognitive effects of WBRT in the elderly population with PCNSL, it should not be forgotten that WBRT maintains an important palliative role in patients achieving partial response, or who are not candidate for hd-MTX based chemotherapy. Patients unfit for a protracted course of RT can be offered low-dose WBRT or a course of hypo-fractionated RT (e.g. 30 Gy/10 fractions). For very elderly patients who are too confused to undergo RT safely, palliative management with steroids alone may be the preferred option.

## Brain metastases

Brain metastases occur in up to 40% of patients with cancer, and treatment options include supportive care, surgery and RT. WBRT has classically been the standard treatment for patients with brain metastases with a reported median survival of 3–6 months [92]. Older age has been reported as an unfavorable prognostic factor for survival [93–95]; using recursive portioning analysis (RPA) the Radiation Therapy Oncology Group (RTOG) has analyzed 1200 patients treated with WBRT enrolled in three consecutive RTOG trials conducted between 1979 and 1993, describing three prognostic classes defined by age, KPS, and disease status [93]. The reported survival for patients of 65 years and over (RPA class II and III) was less than 5 months, with the worst outcome observed in patients with a KPS < 70 (RPA class III). In addition, the use of WBRT has been associated with the risk of neurocognitive deterioration [96–99], and this is of concern especially in older patients.

SRS has been increasingly used in the initial management of patients with brain metastases. The rationale for this approach is to achieve local control while avoiding the risk of the detrimental neurocognitive effects of WBRT. Although no prospective trials have been specifically addressed the clinical outcomes of SRS in older patients with brain metastases, data extracted from three randomized studies comparing the use of WBRT plus SRS versus SRS alone in patients of all ages show no significant survival differences between younger and older patients [99–103]. Aoyama et al. [100] reported 132 patients with 1 to 4 brain metastases randomized to receive WBRT plus SRS or SRS alone. The use of SRS alone resulted in a similar survival and risk of neurologic death as compared with WBRT plus SRS, with no significant differences between patients < 65 years and those ≥ 65 years. In another randomized trial of 213 patients with 1 to 3 brain metastases treated with SRS or SRS plus WBRT between February 2002 and December 2013, Brown et al. [103] reported similar median overall survival times of 7.4 months for patients who received SRS plus WBRT and 10.4 months for those receiving SRS alone (hazard ratio 1.02; 95% CI, 0.75–1.38; *p* = 0.9). Analysis by age, extracranial disease status, and number of brain metastases revealed no survival benefit in any group. Similar overall survival times in the range of 6 to 12 months without a clinically significant neurocognitive decline have been reported in few retrospective series of older patients with brain metastases treated with SRS [104–106].

Interestingly, the recently proposed diagnosis-specific graded prognostic assessment (DS-GPA) score offers different patterns of diagnosis-specific prognostic factors and seems more appropriate than RPA Classes in predicting the outcome that can be expected from the various treatment options in the elderly population [107]. According to the DS-GPA scores, number of metastases and/or KPS, but not age, are significant prognostic factors for survival in several types of cancer, including breast cancer, renal cell cancer, gastrointestinal cancer, and melanoma.

The main reason for using SRS alone is that WBRT may be associated with a decline in quality of life [108, 109] and neurocognitive function [98, 99, 101, 103]. In 58 patients with 1 to 3 metastases randomly assigned to receive WBRT plus SRS or SRS alone, Chang et al. [101] observed that patients treated with SRS plus WBRT were significantly more likely to show a decline in learning and memory function at 4 months than patients assigned to receive SRS alone. In another randomized trial comparing the cognitive function in patients treated with SRS alone or SRS plus WBRT, Brown et al. [103] observed significantly less cognitive deterioration at 3 months after SRS alone as compared with SRS plus WBRT (difference, −28.2%; 90%CI, −41.9% to −14.4%; *p* < .001). In addition, overall quality of life was higher at 3 months after SRS alone (mean change from baseline, −0.1 vs −12.0 points; mean difference, 11.9; 95%CI, 4.8–19.0 points; *p* = 0.001).

Two recent studies have evaluated the protective effects of memantine and IMRT planning for hippocampus sparing on cognitive function among patients receiving WBRT for brain metastases [110, 111]. In the phase III RTOG 0614, 508 adult patients were randomized to receive WBRT with placebo or memantine (20 mg/d) for 24 weeks [110]. The study failed to demonstrate a significant less decline in delayed recall in the memantine arm (*p* = 0.059), possibly due to the low number of analyzable patients at 24 weeks; however, patients receiving memantine had better cognitive function over time as compared with those receiving WBRT alone; specifically, memantine delayed time to cognitive decline and reduced the rate of decline in memory, executive function, and processing speed. In the RTOG 0933 single-arm phase II study of 113 patients who received WBRT with hippocampal sparing for brain metastases, results of cognitive function and health-related QOL, assessed by the Hopkins Verbal Learning Test–Revised Delayed Recall and the Functional Assessment of Cancer Therapy–Brain subscale (FACT-BR), respectively, were compared with those observed in prespecified historical control of patients treated with standard WBRT (30 Gy in 10 fractions) [111]. Among 42 patients who were analyzable at 4 months, mean relative decline in delayed recall from baseline to 4 months was 7.0% (95% CI, −4.7 to 18.7%), being significantly lower than historical control (*p* < .001); no decline in QOL scores was observed. Based on these results, a randomized phase III trial exploring the use of memantine and WBRT with or without hippocampal avoidance in patients with brain metastases has been activated (https://clinicaltrials.gov/ct2/show/NCT02360215).

In summary, data extracted from randomized trials and retrospective studies suggest that SRS is a reasonable approach to older patients with a limited number of brain metastases with both survival benefit and toxicity profile similar to those observed in young adults. Future randomized studies need to investigate the advantages of such approach in the elderly in terms of survival and quality of life over other treatment options.

## Benign tumors

Incidence of benign tumors, including meningiomas, acoustic neuromas and pituitary adenomas increases with age [4]. Meningiomas constitute the most common non-glial brain tumor histological type and accounts for approximately 12–20% of all primary intracranial tumors. The risk for developing meningioma grows with age and increases dramatically after the age of 65, reaching a peak at the seventh decade of life. An incidence of 8.5 per 100,000 persons per year has been recorded among elderly people, which is significantly higher compared to 1–2.8 cases per 100,000 persons per year estimated for the general population [4, 112]. While surgery has traditionally been the mainstay of treatment of symptomatic and fast growing tumors in all age groups, RT is frequently employed after incomplete resection, recurrent tumors, or for patients at risk of severe morbidity with a reported excellent local control and low toxicity [113].

In general, large published series including patients of all ages with a meningioma treated with either SRS or fractionated stereotactic RT (FSRT) reported no differences in local control and treatment-related toxicity between younger and older patients [114–121]. Two retrospective studies have assessed the outcome of FSRT in older patients with meningiomas [122, 123]. In a series of 121 patients treated with FSRT (55.8 Gy in 1.8 Gy fractions), hypofractionated stereotactic RT (25-35Gy in 5–7 fractions) or SRS (15–18 Gy), Fokas et al. [122] reported a similar local control of about 95% at 5 years, with no new neurologic deficits, radiation necrosis or radiation-induced secondary malignancies. In another study of 100 patients aged 65 or older (median age 71 years) treated with FSRT (56.5 Gy), hypofractionated stereotactic RT (36.3 Gy in 5–7 fractions) or single-fraction SRS (17.6 Gy), Kaul et al. [123] observed a 5-year local control of 91.1%, with no grade 2 or 3 neurological toxicity. No study have specifically addressed the outcome of RT in older patients with either secreting or nonsecreting pituitary adenomas, and acoustic neuromas; however, data reported in large retrospective studies and systematic reviews show similar local control and toxicity between young and older patients after either SRS or FSRT [124–142]. Single-fractions doses of 13–16 Gy and 20–28 Gy are usually employed for non-functioning and secreting pituitary adenomas [124, 125, 129–134], respectively, and of 12–14 Gy for acoustic neuromas [135, 137, 139–142]. Hypofractionated RT and FSRT using doses of 21–25 Gy in 3–5 fractions and 45–54 Gy in 25–30 daily fractions of 1.8 Gy, respectively, are frequently employed for large tumors involving the optic pathway or compressing the brainstem [126–128, 133–136, 138].

## Conclusions

RT remains an effective treatment in elderly patients with brain tumors. For large malignant gliomas, randomized studies comparing standard RT versus hypofractionated RT show similar survival benefit, although short-term courses of RT are associated with a better safety profile. Decisions regarding the choice between RT and TMZ chemotherapy should be based on the assessment of MGMT promoter methylation status. Patients with methylated tumors receive the most significant survival benefit from treatment with TMZ; by contrast, chemotherapy produces no benefit in patients with unmethylated tumors, suggesting that RT is a better option in these patients. An abbreviated course of RT plus TMZ has recently emerged as a safe treatment associated with improved survival over RT alone.

SRS alone represents a feasible approach for older patients with a limited number of brain metastases, with reported survival and risk of neurologic death similar to those observed for younger patients. This approach allows omitting or the delaying the use of WBRT in older patients, who are usually more sensitive to the negative impact of cranial irradiation on neurocognitive function and quality of life. Similarly, the use of stereotactic techniques, either SRS or FSRT, has permitted the delivery of safe radiation doses in older patients with skull base tumors, leading to excellent long-term tumor control with minimal side effects and preservation of quality of life. The choice of stereotactic technique is usually based on size and location of tumor. In clinical practice, SRS is recommended for small-to-moderate tumors (<2.5-3 cm) that do not involve radiosensitive structures, such as optic chiasm and brainstem; hypofractionated SRT or FSRT would be a better treatment option when a single-fraction dose carries an unacceptable risk of toxicity.

Future studies need to evaluate the impact of different radiation techniques on survival, neurocognitive outcome and quality of life in older patients with brain tumors, as well their comparison with regimens incorporating RT and/or chemotherapy. A rigorous assessment of tolerance of different brain structures, including optic chiasm, cranial nerves, brainstem, and hippocampus to different radiation dose/fractionation schedules in older population is a research priority for radiation oncologists.
